# Altered circadian rhythmicity of the QT interval predicts mortality in a large real-world academic hospital population

**DOI:** 10.1016/j.heliyon.2024.e41308

**Published:** 2024-12-19

**Authors:** Rutger R. van de Leur, Bastiaan C. du Pré, Markella I. Printezi, Rutger J. Hassink, Pieter A. Doevendans, René van Es, Linda W. van Laake

**Affiliations:** a Department of Cardiology, University Medical Center Utrecht, Utrecht, the Netherlands; b Department of Internal Medicine, Erasmus Medical Center, Rotterdam, the Netherlands; c Netherlands Heart Institute, Utrecht, the Netherlands; d Central Military Hospital, Utrecht, the Netherlands; e Utrecht University, Utrecht, the Netherlands

**Keywords:** Electrocardiography, QT interval, Circadian rhythm

## Abstract

**Objective and rationale:**

Small studies have shown that the QT interval follows a circadian rhythm. This finding has never been confirmed in a large real-world hospital population and the clinical meaning of disrupted rhythmicity remains unknown.

**Methods:**

In this cohort study, all consecutive adult patients with at least one 12-lead ECG acquired between 1991 and 2021 were considered. Sinus rhythm ECGs without QRS conduction or ST-segment abnormalities obtained at the wards or outpatient clinic were included. The QT interval was corrected for age, sex and ventricular rate in a personalized manner. Subsequently, the added value of a 24-h sinusoid of time-of-day was evaluated. An individual 24-h QT interval amplitude was obtained from the model in a subset with patients that had at least 3 ECGs of which one during the night before their last ECG. The association of this individual QT interval with all-cause mortality was assessed using a left-truncated Cox regression model.

**Results:**

The baseline QT correction model was fitted using 237,555 ECGs of 100,644 patients. The personalized corrected QT interval had no relationship with ventricular rate (*r* = −0.008). Adding the 24-h sinusoidal to the baseline model resulted in a significantly better fit (p < 0.0001). The mean circadian variation of the QT interval was 15 ms, with the maximum QT duration around midnight and an effect that is largest in young female patients. A non-linear relationship between peak-to-trough amplitude in QT interval rhythmicity and all-cause mortality was found, with both lower and higher values associated with increased risk.

**Conclusions:**

Using heterogeneous, real-world hospital data of more than 100,000 patients, circadian rhythmicity proved to be an independent determinant of the QT interval. Both increased and diminished QT rhythmicity was shown to be a predictor of all-cause mortality. QT interval should be corrected for the time-of-day and altered circadian rhythmicity should trigger awareness of increased mortality risk (https://qt.ecgx.ai).

## Highlights

• Our findings confirm results of small studies describing circadian rhythms in QT interval in a large and heterogeneous population.
• Circadian rhythmicity was an independent determinant of corrected QT interval; the effect was largest in young female patients.
• Altered individual QT interval rhythmicity was a predictor of all-cause mortality.
• A personalized corrected QT interval and QT rhythmicity can be easily calculated via https://qt.ecgx.ai.

## 1 Introduction

The QT interval is an ECG parameter for ventricular repolarization that is widely used in clinical practice. Both short and long QT interval are associated with ventricular arrythmias [1]. The QT interval is determined by various factors such as ventricular rate, age, sex, genetics, medication use, and time-of-day [2]. Previous small studies found that the QT interval peaks around midnight and that the circadian (day-night, diurnal or 24-h) rhythm in QT interval is governed by an intrinsic, molecular circadian clock that regulates expression of cardiac ion channels [[3], [4], [5]]. Formally, the qualification circadian applies to intrinsic, autonomous rhythms only, but in practice the term is used for all 24-h rhythms regardless of the presence of external cues. Preclinical and small clinical studies demonstrate that disruption of heart rate corrected QT interval (QTc) rhythmicity, for example after myocardial infarction, is associated with ventricular arrythmias [3,6,7]. However, circadian variation in QT interval has not been studied in a large, heterogeneous, real-world population. In the current study, we investigate circadian QT rhythmicity and its association with mortality in a large academic population using a database of almost 1 million ECGs.

## 2 Methods

### 2.1 Study participants and data acquisition

The cohort consisted of all patients between 18 and 85 years that had at least one 10 s 12-lead ECG acquired between July 1991 and August 2021 in the University Medical Center Utrecht (UMCU). The study was approved by the UMCU ethical committee with number 18–827. As all data were deidentified, written informed consent was waived by the UMCU ethical committee. Demographic and mortality data for each were extracted from the digital patient records. For all ECGs, ventricular frequency, conduction intervals, hospital department and date and time of acquisition and interpretation by either an overreading physician or the computerized algorithm were extracted from the MUSE ECG system (MUSE version 8; GE Healthcare, Chicago, IL, USA). The conduction intervals were automatically computed using the MUSE 12SL algorithm (different versions, ranging from 16 to 24) [8]. Diagnostic statements were extracted from the free text interpretation of the ECG by a physician, if available, or by the computerized interpretation algorithm using a text mining-based approach described before [9]. These statements also mentioned whether the ECG was technically adequate or whether lead reversal was present.

As QT measurements can be unreliable for some ECG abnormalities, only technically adequate sinus rhythm ECGs without any AV or QRS conduction abnormalities (eg. second- and third-degree AV block, non-specified intraventricular conduction delay, right and left bundle branch block or fascicular blocks) or ST-segment abnormalities (eg. nonspecific ST-segment abnormalities, negative T waves, ST depression or elevation) were included. Moreover, ECGs with outliers in the measured ventricular frequency (below 25 or above 175 beats per minute) and corrected QT interval (below 200 or above 600 ms) were excluded. Finally, ECGs obtained at hospital departments where many external factors could influence the QT interval (eg. intensive care unit (ICU), emergency department (ED) and cardiac care unit (CCU)) were excluded.

### 2.2 Primary and secondary analysis

In the primary analysis, the QT interval was modelled in relationship to the sinusoidal of the time-of-day using the complete dataset. For the secondary analysis, where the association between circadian rhythms and mortality was investigated, a subset of participants was used that had a least 3 ECGs (of which one between 6 p.m. and 6 a.m.) within a 3 year window.

### 2.3 Statistical analysis

Baseline characteristics are presented as mean ± SD or median with interquartile range, where appropriate. For the primary analysis, a baseline model that allowed for between-subject variation in the QT interval was fitted to correct the QT interval for age, sex and RR interval. Afterwards, a sinusoidal of the time-of-day (circadian rhythm) was added to this model and was fitted using the trigonometric identity, where a sine wave with amplitude A and phase shift φ is equivalent to the linear combination:

Asin(x+φ)=asin(x)+bcos(x)

and amplitude A and phase shift φ are defined as:

$$A=\sqrt{a^{2}+b^{2}}$$

$$\sin\;\varphi =\frac{b}{\sqrt{a^{2}+b^{2}}}$$

A random intercept per patient was used. Moreover, an autocorrelation structure of order 1 with a continuous time covariate was used as measurements closer in time are more correlated. Non-linear relationships using natural cubic splines and interactions were investigated. Added value of the nonlinearities, interactions and sinusoidal of time-of-day to the model was assessed using Akaike's Information Criterion and likelihood ratio (LR) tests for nested models.

For the secondary analysis, the model was extended to also allow for between-subject circadian variation in the QT amplitude using a random slope. The individual peak-to-trough circadian QT amplitude was subsequently entered into a left-truncated Cox regression model with all-cause mortality as outcome and sex as a covariate. Peak-to-trough circadian QT amplitude was defined as the difference between the longest and shortest modelled QT interval per patient in milliseconds. Age was used as the primary time variable to correct for late-entry as patients had their last ECGs at different ages. Non-linear relationships were assessed using cubic splines.

## 3 Results

**Fig. 1:**
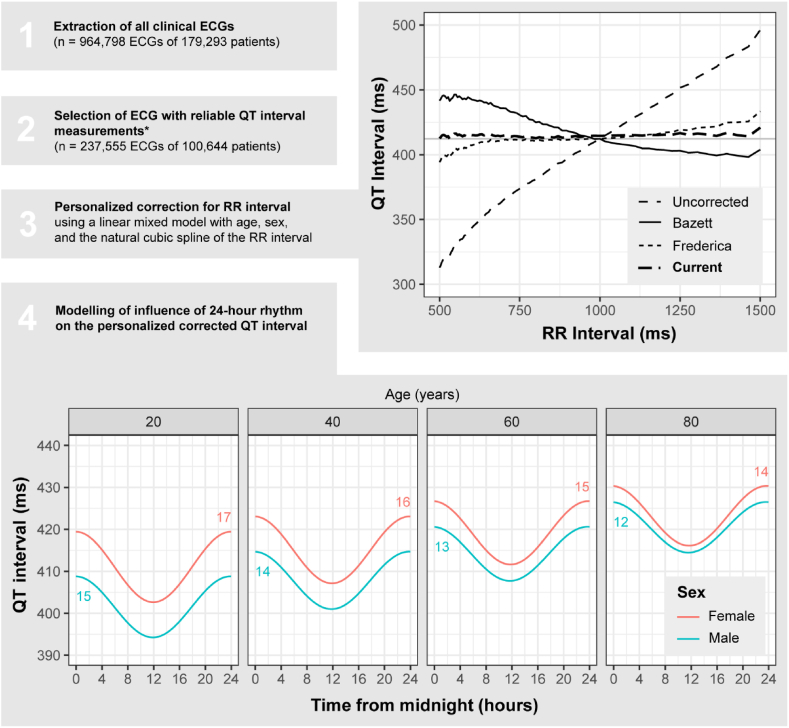
Overview of the methodology, flow of the data and the modelled circadian rhythm in the personalized corrected QT interval of 237,555 ECGs of 100,644 patients, with influence of age and sex shown. The number in part 4 indicates the peak-to-trough for each sex and age combination shown. ∗ECGs with abnormalities that could influence the QT interval measurement (such as ischemia), with outliers in the interval measurements or acquired at the intensive care unit, emergency department and cardiac care unit are excluded. ECG: electrocardiogram, ms: milliseconds.

In total, 954,798 ECGs of 179,293 patients were assessed. Of these 611.160 (64 %) were overread by a physician. After exclusion of the prespecified ECG abnormalities (n = 594,545; 62 %), outliers due to measurement errors (n = 34,199; 3.6 %) and ECGs obtained in the ICU, ED or CCU (n = 88,499; 9.2 %), 237,555 ECGs of 100,644 patients were included (Table 1). The best baseline model for QT interval consisted of a natural cubic spline of the RR interval with knots at 600 ms and 1000 ms, in combination with age, sex and all pairwise interactions. The personalized corrected QT interval from this model showed no relationship with ventricular rate (*r* = −0.008), while known correction methods, such as Bazett, Frederica and Framingham, were inferior in their correction for ventricular rate (r*=*0.43, *r* = −0.14 and *r* = −0.15, respectively; Fig. 1).

**Table 1:** Baseline characteristics for all individual ECGs included in the analysis, stratified for acquisition during day and nighttime. There were no missings.

	Day (6 a.m.–6 p.m.)	Night (6 p.m.–6 a.m.)	p-value
n (%)	226550 (95.4)	11005 (4.6)	
Age at ECG - median [IQR]	61 [49, 70]	61 [48, 71]	0.007
Male sex - n (%)	123779 (54.6)	5939 (54.0)	0.171
Location - n (%)			<0.001
Cardiology outpatient clinic	76313 (33.7)	87 (0.8)	
Cardiology ward	30748 (13.6)	2830 (25.7)	
Non-cardiology outpatient clinic	43002 (19.0)	180 (1.6)	
Non-cardiology ward	33447 (14.8)	7901 (71.8)	
Pre-operative screening	43040 (19.0)	7 (0.1)	
Hours since midnight - n (%)			<0.001
0 - 3	–	1552 (14.1)	
3 - 6	–	1140 (10.4)	
6 - 9	18348 (8.1)	–	
9 - 12	99002 (43.7)	–	
12 - 15	76315 (33.7)	–	
15 - 18	32884 (14.5)	–	
18 - 21	–	4774 (43.4)	
21 - 24	–	3539 (32.2)	
Ventricular rate - median [IQR]	70 [61, 81]	80 [67, 97]	<0.001
PR interval - median [IQR]	158 [142, 176]	152 [136, 172]	<0.001
QRS duration - median [IQR]	92 [84, 100]	88 [82, 98]	<0.001
QT interval - median [IQR]	388 [366, 412]	380 [348, 410]	<0.001
QTc Bazett - median [IQR]	418 [404, 437]	435 [416, 454]	<0.001
QTc Fredericia - median [IQR]	409 [396, 424]	414 [398, 433]	<0.001
QTc Framingham - median [IQR]	409 [396, 423]	414 [397, 431]	<0.001

Adding the sinusoidal of the time-of-day into the model resulted in a significantly better fit (LR 2372, p < 0.0001), and adding interactions between time-of-day and the other variables in the baseline model improved the fit further (LR 871, p < 0.0001; Supplementary Table 1). The model showed that the QT interval peak-to-trough amplitude was highest for females and decreased with age. Overall, the difference between peak and trough for the average patient (a male of age 60 with ventricular rate of 60 bpm) was 15 ms, with the maximum duration around midnight (Fig. 1). Coefficients of the final model are presented in Supplementary Table 2.

**Fig. 2:**
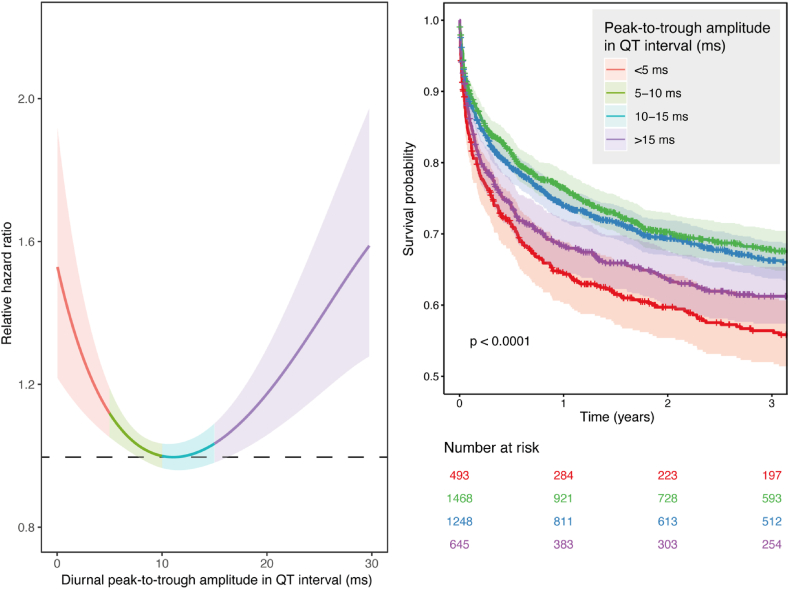
Association of diurnal peak-to-trough amplitude in QT interval with all-cause mortality. For each patient the rhythmicity in QT interval is estimated for all ECGs in a period of three years, and related to all-cause mortality using Cox regression. This model shows a non-linear relationship, with higher hazards for low amplitudes (<5 ms) and for high amplitudes (>15 ms).

For the secondary analysis, a dataset of 13,704 ECGs of 4019 patients was available. 1446 (36 %) patients died during a median follow-up of 4.8 years [IQR 1.8–8.6 years]. Left-truncated Cox regression showed a non-linear significant relationship between peak-to-trough amplitude in QT interval diurnality and mortality. Both lower and higher amplitudes were associated with increased all-cause mortality risk. Patients with a peak-to-trough amplitude around 10 ms had the lowest risk (Fig. 2).

## 4 Discussion

In this study, we investigated circadian rhythmicity of the QT interval in an academic hospital population. Using data of more than 100,000 patients, we confirmed that QT interval duration is determined by several factors such as heart rate, age, sex, and time-of-day. In a multivariate model, time-of-day proved to be an independent determinant of the QT interval, with an effect that is largest in young female patients. In the studied population, mortality risk was higher in patients with increased or diminished QT rhythmicity.

Our findings are in line with several studies which found circadian rhythms in QT interval and other ventricular repolarization parameters independent of heart rate, but adds three important things [3,7,[10], [11], [12], [13]]. First, previous research was done in healthy volunteers or small groups of specific patients. We show that QT rhythmicity is present in a large, heterogeneous population. Second, previous studies used specific methods to correct the QT interval for heart rate. Smetana et al. showed that the extent of circadian variation strongly depends on the method used [5]. By developing a personalized correction that outperforms standard methods in the full heart frequency range, we ascertain that our results are not caused by inadequate correction for heart rate. Third, previous studies showed conflicting results regarding mortality; some studies found that reduced QT rhythmicity is associated with mortality, while others report the opposite.^3,7,14^ We clarify this paradox by showing that the association of QT interval duration and mortality risk is U-shaped: both increased and decreased QT rhythmicity are associated with mortality.

Our dataset is large and consists of a heterogeneous patient population. We were therefore able to develop a statistically sound QT prediction model which allows for precise correction of heart rate and other variables in a broad, clinically relevant setting. Using our online tool, an accurate, personalized corrected QT interval and QT rhythmicity can be easily calculated and used to detect aberrant ventricular repolarization (https://qt.ecgx.ai).

The study has several limitations. Firstly, only all-cause mortality was available to investigate the relationship between circadian rhythm and outcomes. Stronger conclusions could have been drawn with cause-specific mortality and more information on comorbidities. Secondly, we had to rely on automated measurement of the QT interval using different software versions and had no information on medication use that could influence the QT interval. To mitigate this, we excluded many ECGs with abnormalities that influence accurate QT measurement and excluded ECGs from departments where medication influencing the QT interval is often administered (i.e. the cardiac care unit and intensive care unit). This makes that the results are not generalizable to ECGs with these abnormalities or made at these hospital departments. Finally, evaluation of the circadian QT correction model on an external test set should be performed to confirm its generalizability.

In conclusion, circadian rhythmicity proved to be an independent determinant of the QT interval in a heterogeneous, large, real-world dataset. Both increased and diminished QT rhythmicity was shown to be a predictor of all-cause mortality. QT interval in clinical practice should be corrected for the time-of-day, and altered circadian rhythmicity should trigger awareness of increased mortality risk.

## CRediT authorship contribution statement

**Rutger R. van de Leur:** Writing – original draft, Visualization, Software, Methodology, Investigation, Formal analysis, Data curation, Conceptualization. **Bastiaan C. du Pré:** Writing – review & editing, Validation, Supervision, Conceptualization. **Markella I. Printezi:** Writing – review & editing, Methodology. **Rutger J. Hassink:** Writing – review & editing, Supervision, Funding acquisition. **Pieter A. Doevendans:** Writing – review & editing, Supervision, Project administration, Funding acquisition, Conceptualization. **René van Es:** Writing – review & editing, Validation, Supervision, Software, Project administration, Methodology, Investigation, Formal analysis, Data curation, Conceptualization. **Linda W. van Laake:** Writing – review & editing, Supervision, Funding acquisition, Conceptualization.

## Ethical approval statement

The study was approved by the UMCU ethical committee with number 18–827. As all data were deidentified, written informed consent was waived by the UMCU ethical committee.

## Data availability

The datasets used in this study are not openly available due to privacy concerns.

## Funding

This study was financed by the 10.13039/501100001826Netherlands Organisation for Health Research and Development (10.13039/501100001826ZonMw) with grant number 104021004 and the Dutch Heart Foundation with grant number 2019B011. PD is supported by Psider-heart and Leducq CUREPLaN and PRIORITY.

## Declaration of competing interest

The authors declare the following financial interests/personal relationships which may be considered as potential competing interests: RvdL and RvE are cofounders, shareholders and board members of Cordys Analytics B.V., a spin-off of the UMC Utrecht that has licensed AI-ECG algorithms. PD is founder and shareholder of HeartEye B.V., an ECG-device company. If there are other authors, they declare that they have no known competing financial interests or personal relationships that could have appeared to influence the work reported in this paper.
