# Supplementary material for: Altered circadian rhythmicity of the QT interval predicts mortality in a large real-world academic hospital population
Source: Heliyon. 2024 Dec 19;11(1):e41308. doi: 10.1016/j.heliyon.2024.e41308 (PMC11731474; doi:10.1016/j.heliyon.2024.e41308)
Supplement: Multimedia component 1 [file mmc1.docx]

**Supplemental Tables**

| Formula | AIC | Likelihood ratio | LRT p-value |
| --- | --- | --- | --- |
| Ventricular rate | 2070953 |  |  |
| Natural cubic spline of ventricular rate | 2062218 | 8738 | <0.0001 |
| Above and age and sex | 2056606 | 5617 | <0.0001 |
| Above and all interactions | 2055957 | 657 | <0.0001 |
| Above and sinusoidal of time-of-day | 2053470 | 2491 | <0.0001 |
| Above and all interactions | 2053096 | 387 | <0.0001 |

*Supplemental Table 1.* Likelihood ratio test and Akaike’s Information Criterion for different QT correction methods tested. First, a model that only corrected the QT interval for a linear relation with the ventricular rate was used as a baseline. Then additional variables were added in a nested manner. Added value of the additional variables was tested using the likelihood ratio test. AIC: Akaike’s Information Criterion, LRT: likelihood ratio test.

| Variable | Coefficient | p-value |
| --- | --- | --- |
| Intercept | 257 | <0.001 |
| Male sex | 1.44 | 0.19 |
| Age per year | 0.20 | <0.001 |
| Natural cubic spline of ventricular rate (first piecewise polynomial) | 153 | <0.001 |
| Natural cubic spline of ventricular rate (second piecewise polynomial) | 358 | <0.001 |
| Natural cubic spline of ventricular rate (third piecewise polynomial) | 293 | <0.001 |
| cos(2 * pi * Time) | 7.52 | <0.001 |
| sin(2 * pi * Time) | -0.43 | <0.001 |
| Male sex:age per year | 0.11 | <0.001 |
| Male sex:natural cubic spline of ventricular rate (first piecewise polynomial) | -10.2 | <0.001 |
| Male sex:natural cubic spline of ventricular rate (second piecewise polynomial) | -9.62 | 0.002 |
| Male sex:natural cubic spline of ventricular rate (third piecewise polynomial) | -7.53 | 0.01 |
| Male sex:cos(2 * pi * Time) | -1.11 | <0.001 |
| Male sex:sin(2 * pi * Time) | -0.078 | 0.57 |
| Age per year:cos(2 * pi * Time) | -0.022 | 0.001 |
| Age per year:sin(2 * pi * Time) | -0.0074 | 0.09 |
| Ventricular rate:cos(2 * pi * Time) | 9.06 | <0.001 |
| Ventricular rate:sin(2 * pi * Time) | -1.65 | <0.001 |

*Supplemental Table 2.* Coefficients of the final linear mixed effects model for prediction of QT interval, including a natural cubic spline of the ventricular rate, age, sex, the sinusoidal of time-of-day and all pairwise interactions.
